# Barriers and facilitators to cervical cancer screening among under-screened women in Cuenca, Ecuador: the perspectives of women and health professionals

**DOI:** 10.1186/s12889-022-14601-y

**Published:** 2022-11-22

**Authors:** Bernardo Vega Crespo, Vivian Alejandra Neira, José Ortíz Segarra, Andrés Andrade, Gabriela Guerra, Stalin Ortiz, Antonieta Flores, Lorena Mora, Veronique Verhoeven, Ana Gama, Sónia Dias, Bo Verberckmoes, Heleen Vermandere, Kristien Michelsen, Olivier Degomme

**Affiliations:** 1 Facultad de Ciencias Médicas, Universidad de Cuenca, 010203 Cuenca, Ecuador; 2 Facultad de Medicina, Universidad del Azuay UDA, Cuenca, 010104 Ecuador; 3 Family Medicine and Population Health, University of Antwerp, 2610 Antwerp, Belgium; 4 NOVA National School of Public Health, Public Health Research Centre, Universidade NOVA de Lisboa, Lisbon, 1169-056 Portugal; 5 Comprehensive Health Research Center (CHRC), Lisbon, 1169-056 Portugal; 6 International Centre for Reproductive Health (ICRH), Department of Public Health and Primary Care, Faculty of Medicine and Health Sciences, Ghent University, Ghent, 9000 Belgium

**Keywords:** Cervical cancer, Screening, Barriers and facilitators, Ecuador, HPV

## Abstract

**Background:**

Cervical cancer screening is a cost-effective method responsible for reducing cervical cancer-related mortality by 70% in countries that have achieved high coverage through nationwide screening strategies. However, there are disparities in access to screening. In Ecuador, although cervical cancer is the second most common cancer in women, only 58.4% of women of reproductive age have ever been screened for cervical cancer.

**Methodology:**

A qualitative study was performed to understand the current barriers to screening and to identify strategies that could increase uptake in Azuay province, Ecuador**.** Seven focus group discussions (FGDs) were conducted with under-screened women and health professionals (HPs). The FGDs were recorded and transcribed. Content analysis was done using the socio-ecological framework to categorize and analyse the data.

**Results:**

Overall, 28 women and 27 HPs participated in the study. The two groups perceived different barriers to cervical cancer screening. The HPs considered barriers to be mainly at the policy level (lack of a structured screening plan; lack of health promotion) and the individual level (lack of risk perception; personal beliefs). The women identified barriers mainly at organizational level, such as long waiting times, lack of access to health centres, and inadequate patient–physician communication. Both groups mentioned facilitators at policy level, such as national campaigns promoting cervical cancer screening, and at community and individual level, including health literacy and women’s empowerment.

**Conclusions:**

The women considered access to health services the main barrier to screening, while the HPs identified a lack of investment in screening programmes and cultural patterns at the community level as major obstacles. To take an integrated approach to cervical cancer prevention, the perspectives of both groups should be taken into account. Additionally, new strategies and technologies, such as self-administered human papillomavirus (HPV) testing and community participation, should be implemented to increase access to cervical cancer screening.

## Introduction

Cervical cancer still represents a considerable threat to women’s health: in 2020, 604,000 new cases were reported worldwide, and more than 341,000 women died from cervical cancer [1]. Almost 90% of these cases occurred in low- and middle-income countries, and the number of deaths is projected to increase by 25% over the next 10 years if comprehensive measures are not taken [2, 3]. Cervical cancer screening is a cost-effective method that has led to a reduction in cervical cancer-related mortality of 70% in countries that have achieved high uptake [4].

In Ecuador, cervical cancer is the second most common cancer in women. In 2020, 1534 new cases were detected, and 813 women died of this cause [5]. The incidence of cervical cancer in Ecuador is 17.8 per 100,000 women. The only country in South America with a higher incidence is Bolivia (35.8 per 100,000 women). Brazil has the lowest incidence in the region (12.2 per 100,000 women) [6]. The number of deaths each year has increased in Ecuador over the last 10 years, which is an indication that current public health policies are insufficient or poorly implemented [6, 7].

Ecuador’s national cervical cancer prevention strategy from 2015 recommends three-yearly screening, based on cytology, for sexually active women between the ages of 21 and 67 years, and offers human papillomavirus (HPV) vaccines to adolescent girls aged 9–12 years [8–10]. However, in some health centres, women are offered yearly screening. Most screening services are offered in public primary health care centres for free, but women can also be screened at non-governmental organization and private health centres at low cost. Only in some public and private services in urban areas is HPV DNA testing offered as primary screening and for follow-up in case of cervical abnormalities [11, 12].

The World Health Organization’s global strategy to accelerate the elimination of cervical cancer suggests that to reduce cervical cancer-related mortality, up to 70% of all women should be screened with a high performance test at least twice during their lifetime, at 35 years and again at 45 years [13]. To reach that goal in terms of screening uptake, barriers to screening should be clearly identified and overcome [14].

Despite the availability of free, public services, only 58.4% of women of reproductive age in Ecuador have ever been screened for cervical cancer during their lifetime. In other words, 41.6% of eligible women in Ecuador have never been screened, which reveals the presence of major limitations in the national screening programme [15–17]. Few publications have ever presented a unique profile of never-screened women in Ecuador, and these have mainly concentrated on large populations with a common characteristic. However, the most commonly reported characteristics related to never having been screened for cervical cancer are living in a remote rural area, belonging to an indigenous community and having a low educational level [17–19].

In Ecuador, only two studies have addressed the low coverage of cervical cancer screening: one focuses on the challenges and opportunities of accessing cervical cancer screening among indigenous populations, whereas the other (from 2009) reports on attitudes towards screening among women in urban and rural areas. Barriers identified included organizational obstacles, reduced access to screening in rural, indigenous communities, and individual and cultural barriers, including fear of gynaecological examination, a lack of risk perception and a lack of knowledge about cervical cancer in general [17, 20]. The aim of this research is to complement and update previous studies, by assessing the perspectives of under-screened women and health care providers regarding barriers and facilitators of cervical cancer screening in Cuenca, Ecuador.

## Methods

### Research design

A qualitative study with a phenomenological approach was performed from April 2020 to March 2021 in Azuay province, Ecuador. Focus group discussions (FGDs) were organized with health staff and under-screened women separately, as this method allows participants to interact with each other, which enriches the information generated [21, 22]. This paper follows the consolidated criteria for reporting qualitative research (COREQ) guidelines for reporting qualitative research [23].

### Recruitment and settings

Due to the COVID-19 pandemic and subsequent lockdown measures in Ecuador, some FDGs were carried out online. To ensure biosafety, face-to-face FDGs were carried out in venues where the researchers could control ventilation and the physical distancing of participants.

#### Focus group discussions with women

Women who had never or rarely been screened for cervical cancer were recruited during gynaecological consultations at the Sociedad de Lucha Contra el Cáncer (SOLCA) hospital in Cuenca and during outreach activities of the SOLCA mobile unit in rural areas. Inclusion criteria were being older than 30 years old, speaking Spanish and not having had a Pap smear test in the last 3 years.

The recruitment of participants was conducted by the project researchers. After verifying the inclusion criteria and providing verbal information about the project, the invitation for participation in the FDG was issued. Table 3 presents information about the settings and participants of FDGs for under-screened women.

#### Focus group discussions with health professionals

Health professionals (HPs) were recruited from SOLCA hospital, the mobile unit of SOLCA and the Hospital del Niño y la Mujer (maternal and children’s hospital) in the municipality of Cuenca. All physicians and nurses working in cervical cancer screening as their main activity at the time were invited to participate.

Recruitment of HPs was done through a written invitation to the directors of SOLCA and the Hospital del Niño y la Mujer. Each hospital director passed an invitation to HPs who met the selection criteria. Table 4 presents information about the settings and participants of FDGs for HPs.

### Data collection

Two FGD guides were developed, one for the women and one for the HPs. Key topics addressed during the discussions were: opinions about or experiences with cervical cancer screening; opinions about national cervical cancer screening practices or programmes; barriers that inhibit screening uptake; and suggestions to address these barriers. The FGDs were conducted by a moderator, and two observers took field notes. In addition, participants were asked to complete a brief socio-demographic questionnaire. Table 1 describes the topics, used in FGDs.

**Table 1:** Topics of FGDs

*Focus group discussions with health professionals*	
• What are your opinions about or your experiences with cervical cancer screening among under screened women?	
• What are your opinions about national cervical cancer screening practices or programs?	
• Are there any gaps in these programs?	
• What do you think are the reasons or barriers that lead [insert the specific hard-to-reach group] to not participate in screening/to not participate enough?	
• How can we reduce the barriers we talked about/how can we increase participation?	
*Focus group discussions with women*	
• What are the reasons that lead women to participate in cervical cancer screening?	
• What are the reasons or barriers that lead women to not participate in screening?	
• How can the barriers we talked about be reduced/how to increase participation in cervical cancer screening?	
• From what you know, how do you describe women’s experiences regarding cervical cancer screening?	

### Data analysis

All interviews were audio-recorded and transcribed. The transcripts were uploaded to Nvivo 12, and content analysis was performed [24]. Data analysis and coding were conducted by the first author (BV) and two experienced researchers (VAN; JO). As the objective of the research was to identify barriers and facilitators to cervical cancer screening, an adapted version of the socio-ecological model of Bronfenbrenner [25] was used to identify factors at five different levels: political, organizational, community, interpersonal and individual. The socio-ecological model could give an overview of interactions between several barriers and facilitators at different levels. It also allows integrated solutions identifying different actors and environmental structures to be found. The political level includes national regulations and funding for the system. The organizational level refers to factors related to the health care system. The community level contains cultural values, beliefs and norms, and population characteristics. The interpersonal level refers to a person’s family and related network, and the individual level encompasses knowledge, attitudes and skills, personal characteristics and behaviour [17, 26–28]. A priori categories were defined based on the literature review and organized according to the socio-ecological model. Other emerging categories were identified by inductive analysis [29, 30]. For this study, the most important quotations related to barriers and facilitators were selected and translated into English.

### Ethical aspects

Each FGD started with a presentation of the aims of the study and ethical aspects, namely that participation in the FGD was voluntary, that participants were free to decline to participate or withdraw at any time, and that their anonymity and data confidentiality were assured. All participants provided written informed consent.

## Results

Overall, 28 women took part in four FGDs, and 27 HPs participated in three FGDs. The average duration of the FGDs was 1 hour and 20 minutes. Almost all the FGDs took place face to face, either in a community setting or at SOLCA hospital. However, one FGD with HPs took place online because of the COVID-19 lockdown in force at that time in Ecuador. Table 2 presents selected socio-demographic characteristics of the women and HPs who participated in the FGDs. Tables 3 and 4 present the general characteristics and locations of the FDGs.

**Table 2:** Socio-demographic characteristics

Characteristic	N (%)
**Rarely screened women** [28]	
**Age**	
30–39 years	6 (21.4)
40–49 years	15 (53.6)
50–59 years	6 (21.4)
60–69 years	1 (3.6)
**Residence**	
Urban	19 (67.9)
Rural	9 (32.1)
**Education**	
None	1 (3.6)
Primary	8 (28.6)
Secondary	7 (25.0)
University	12 (42.9)
**Last Pap smear**	
3–4 years ago	6 (21.4)
5 years ago	6 (21.4)
More than 5 years ago	16 (57.1)
**Time needed to reach a health centre**	
Less than 30 minutes	23 (82.1)
Between 30 minutes and 1 hour	4 (14.3)
More than 1 hour	1 (3.6)
**Health professionals** [27]	
**Gender**	
Male	3 (11.1)
Female	24 (88.9)
**Age**	
25–29 years	6 (22.2)
30–39 years	4 (14.8)
40–49 years	4 (14.8)
50–59 years	11 (40.7)
60 years and more	2 (7.4)
**Role**	
Medical doctor	13 (48.1)
Nurse	11 (40.7)
Midwife	3 (11.1)

**Table 3:** Women’s focus groups

Identification code	Date	Location	Number of participants
FGD 1	2 April 2020	Online	6
FGD 2	13 November 2020	Paute municipal hall	6
FGD 3	26 February 2021	University of Cuenca	8
FGD 4	5 March 2021	University of Cuenca	8

**Table 4:** Health professionals’ focus groups

Identification code	Date	Locus	Number of participants
FGD 1	26 February 2020	Online	2
FGD 2	26 July 2020	SOLCA auditorium	11
FGD 3	4 August 2020	Hospital municipal auditorium	6
FGD 4	27 August 2020	SOLCA auditorium	8

**Fig. 1:**
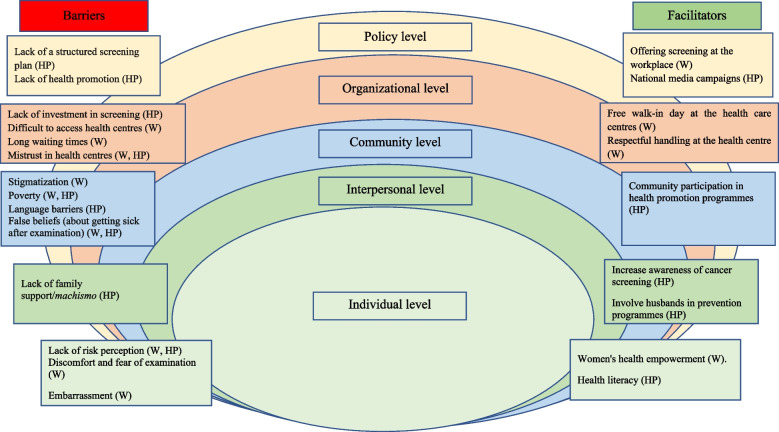
Barriers and facilitators of cervical cancer screening according to the socio-ecological model. Note: W: Women; HP: Health professionals

Results are reported according to the different levels of the socio-ecological model (policy, organizational, community, interpersonal and individual). Both the positive factors (facilitators) and negative determinants (barriers) are mentioned. Finally, suggestions made by study participants to improve uptake of cervical cancer screening based on their positive experiences are presented. Figure 1 presents an overview of the barriers and facilitators at the different levels of the socio-ecological model.

### Individual level

#### Barriers

At the individual level, several main barriers were identified by both the HPs and the women: lack of knowledge, lack of risk perception, lack of time, discomfort and fear. In particular, all participants reported that cervical cancer is not well known or often discussed.

*“Women do not know what a disease like cervical cancer means. That is why they don’t consider Pap smears important... They do not have the correct information ... to be able to get screened. It is an unknown world for many people.” – Medical doctor, 27 years*

*“About cervical cancer … . Not in my family, and neither with my daughter, who is 20 years old. We have not dealt with this issue. None of us are familiar with this issue. The only thing we know is that it is dangerous.” – Woman, 50 years*

A lack of risk perception, caused by a lack of knowledge, means that some women only seek medical attention when they feel symptoms. For many women, feeling healthy is synonymous with being healthy; therefore, they do not see any reason to get screened.

*“I got screened because I felt bad, so I took it, the exam [Pap smear] to see if I had cancer of the uterus.” – Woman, 47 years*

*“There are some adult women who say: ‘I've never had a Pap smear and I'm not going to get it, because I never feel anything and I'm fine and nothing has ever happened to me.’” – Woman, 49 years*

In general, the participants, especially the women, reported a lack of time as a significant barrier. They are expected to first finish all their household obligations before accessing health care. If they work, they need to ask permission to attend a health centre.

*“So we go to the supermarket, we go to work, we pick up the child, we go to the meeting, we go to pay the bills, … and of course, we are busy, we do not have time to get a Pap smear.” – Woman, 49 years*

Women also feel that they do not have the time for screening because they usually have to take care of others first and put their needs last, focusing on the well-being of their children and other priorities in their lives.

*“Culturally, we as women have been left behind – that is, if you get sick, you are the last on the list and you have to heal yourself practically. That is the first mental barrier: we have to think about others and not think about ourselves.” – Woman, 45 years*

Discomfort with the procedure was mentioned as a major barrier by both groups of participants. Several different aspects of discomfort were identified, the first of which was related to the fear of feeling pain during the examination.

*“When that machine [speculum] is introduced, it hurts there, and ... I don't even know why, those things have always hurt me.” – Woman, 62 years*

Another discomfort they mentioned was embarrassment. Women perceived cervical cancer screening as an uncomfortable and even denigrating experience.

*“I think it's like one of the most degrading exams that one can have. It does not feel good. Yes, like degrading, uncomfortable. I think that sometimes it doesn't even hurt, but [it is uncomfortable] because of the tension and stress.” – Woman, 48 years*

For some women, it is even more embarrassing if a male doctor performs the gynaecological examination: they would feel more comfortable if a female doctor were to do it.

*“Maybe I can say that I would have more confidence with a female doctor because when it’s a male doctor, … I don't know. No, well, speaking for myself, since I live alone, I always get nervous when I am going to see a male doctor.” – Woman, 62 years*

Participants even reported that they fear being mistreated or sexually abused during the consultation. Some women mentioned that some doctors flirt during consultations, while others even expressed fear of being raped.

*“I have heard from friends that the gynaecologist, when he takes the Pap smears, touches her and puts his fingers in on purpose, that he does it roughly on purpose and that she feels that it gives him a certain pleasure … I saw him when he put his fingers in: he closed his eyes and yuck, that is horrible, and my husband was not there because he [the doctor] told him to stay out.” – Woman, 49 years*

In addition, women reported poor communication between patients and physicians, claiming that some HPs do not take enough time to explain the procedure step by step. As a result, they do not understand the procedures and even fear them.

*“The doctor did not explain or anything. He told me, ‘Well, go and lie down [on the gynaecological chair], and I'll do it [take the sample].’ From then on I was afraid to go. I said, ‘Now I'm not going to do anything again’, because it was horrible.” – Woman, 49 years*

Finally, some women expressed fear of a possible positive result. Consequently, some women prefer not to be screened, ignoring the fact that early detection could save lives.

*“For fear of the result. She had never had a Pap smear in her life until now, because of fear of the result. [Imagine that] suddenly [the doctor is] saying that she is going to die!" – Woman, 33 years*

#### Facilitators and suggestions for improvement

Empowering women to take control over their own health was considered important to improve uptake of cervical cancer screening.

*“When you love yourself, you take care of yourself, you protect yourself, and you don't expect anyone to protect you as you protect yourself.” – Woman, 49 years*

All HPs agreed that improving women’s health literacy is a priority to improve their knowledge and awareness regarding cervical cancer prevention. Sensitization should start at school and will motivate women to get screened.

*“I think that with education and motivation it should start even at school and then at college and, well, at university.” – Medical doctor, 78 years*

### Interpersonal level

#### Barriers

The lack of family support was considered an important barrier to cervical cancer screening. HPs reported that in some traditional rural communities, women need to ask their husband’s permission to get a gynaecological examination. This *machismo* (sexism) prevents women from getting screened, since men are often reluctant and women follow their advice.

*“A woman of about 42 years old says, ‘I don't know, doctor, because my husband does not want me to do it.’” – Medical doctor, 40 years*

Another reason why men might not approve is because sexually transmitted infections such as HPV could be detected during the medical consultation; this could be a proof of infidelity, for both men and women, and lead to divorce.

*“When she had a positive human papillomavirus test, ... she was obviously a person of a certain level [of education], she asked, ‘How is this transmitted?’ And the doctor told her how it was transmitted, and she said [to her husband], ‘I want a divorce.’ It was like that. The doctor tried to explain, and calm her down, but the patient reacted like that … I will never forget.” – Nurse, 52 years*

#### Facilitators

Facilitators linked to providing information were reported most commonly by HPs. They considered that increasing awareness of cervical cancer screening in communities and households will increase the acceptance of Pap smears and reduce the stigma. In particular, targeting husbands in preventive programmes could improve uptake of cervical cancer screening, since it would help break down barriers caused by the lack of knowledge regarding screening and the *machismo* in those communities.

*“I say, ‘Bring him here [to the health centre] to convince him’, like a joke. I say, ‘Bring him to convince him’ [the husband].” – Medical doctor, 40 years*

### Community level

#### Barriers

Cultural beliefs, even if they are based on misconceptions, constitute a barrier to examinations, as, for example, some women are afraid of getting sick or becoming infected during screening. Some women had experienced bleeding after having a Pap smear; therefore, they suspected that the material used in the procedure was not sterilized well. One participant even mentioned experiencing symptoms of a sexually transmitted infection after the screening.

*“I was fine, and two days after I returned from the exam, it began to feel bad, … [I felt] itching, then a very ugly discharge. I got a very strong infection.” – Woman, 44 years*

According to HPs, women who live in rural areas are screened less often due to the great distance they have to travel to reach health centres. They also believe that these women might be more afraid of having a Pap smear.

*“In rural areas people are still afraid of going to the doctor to have a sample taken.” – Medical doctor, 61 years*

In general, stigma related to Pap smears is a significant barrier, as some women thought that screening is mainly for women who have multiple sexual partners, such as sex workers, or for women who are victims of sexual abuse. As such, getting screened is not considered necessary for a ‘respectable woman’, and participating in regular screening could lead to discrimination by the community.

*“Maybe sex workers [should go for routine screening] because they are in more contact with their clients, let’s say. For their safety they should, they should ... And women or adolescents who are already sexually active ... or girls who are raped, girls who are raped should also already have a Pap test.” – Woman, 56 years*

Overall, some women believed that the main cause of the lack of adherence to routine screening is social inequity. Women who live in poverty, with a low educational level, have less access to screening.

*“Social inequalities exclude more women, and women who are socially excluded – those who we consider more vulnerable in society – have less access to screening tests.” – Woman, 40 years*

Moreover, Spanish is not the first language in some rural communities. HPs recognized that they themselves are not fluent in the local languages; hence poor communication is a bigger problem in these areas.

*“In my indigenous community, we should look for possibilities to do [promotion] in Quichua [an indigenous language], because we try to involve them a lot, although I do not speak it [Quichua] 100 per cent.” – Medical doctor, 40 years*

#### Facilitators and suggestions for improvement

According to the HPs, including the community in health promotion activities – for example, through peer education or disseminating flyers in local languages – would not only increase knowledge about cervical cancer screening but would also help increase its acceptance.

*“A great advantage in our community is that health promoters are indigenous, so they do not have language barriers, so they can help us with this type of promotion. We have even used some brochures in Quichua.” – Medical doctor 40 years*

Also, the support of political and religious leaders in the community could help spread positive messages and might encourage the population to attend screening. Participants considered young community leaders more open to collaborating with this type of health promotion.

*“We involved leaders. Some of them are young and very open to promote this type of procedure.” – Medical doctor, 40 years*

*“Talking with the priest. Maybe if the priest talks in the same church [during a religious service] it would be spectacular.” – Nurse, 52 years*

### Organizational level

#### Barriers

HPs and women reported barriers related to both access to health centres and the quality of the health care services provided. In particular, they identified difficulties and challenges at every step of the cervical cancer screening process: from making the appointment to obtaining follow-up care.

Getting an appointment was described as a major difficulty, since the waiting time can be up to 3 months. In addition, once an appointment is made, it can still be changed, and women only find out when they arrive for their appointment on the day originally scheduled. As a result, many women are discouraged from returning to the health centre.

*“I was going to my appointment one day, but they gave me another appointment for the next day, and then again [the next day]. They said, ‘Come tomorrow.’ I kept going all week, but no, [every time] they gave me an appointment for another day.” – Woman, 41 years*

*“If you call and they answer, you are lucky. If not, you spend a week calling and they never answer. If you are able to make an appointment, they give you one in three months: ‘Come on in on that date’, and so we go that day, and sometimes the doctor has gone on vacation, and there is no one to replace him.” – Woman, 41 years*

Because of these problems, some women opt for cervical cancer screening at private clinics or non-governmental organizations. However, both the HPs and the women reported that due to the cost this is only accessible for those women who can afford this type of care.

*“Because in the countryside it is very complicated for the mothers, so they are selling eggs or exchanging things to get money … They have to see how they can support the family. If they have to pay for their [cervical cancer screening] test and it has a very high cost, they do not go.” – Nurse, 54 years*

The women also reported that the COVID-19 pandemic increased waiting times. In addition, they considered that HPs prioritized COVID patients, which made it is even more difficult to get an appointment at that time.

*“If you get sick right now, they tell you, ‘Go to the health centre.’ But they don't attend to you. Right now it's more – because of COVID – ‘Go, or call the hospital’, yet they don't attend to you.” – Woman, 41 years*

According to the women, the waiting does not end after getting an appointment. Once they have an appointment, they spend an entire morning at the health centre actually getting screened.

*“Going to have a Pap smear at a health centre takes all day! The whole morning. People just don’t have that time.” – Woman, 47 years*

After the sample is taken, patients wait another one or 2 months, which is the minimum time it takes to process the samples and share the results with the health centre. Participants thought that this delay compromises treatment if cancer is found.

*“The result is given after three months.” A Pap smear is taken, and they say, ‘Come back for the results. You can come in about three months.’ And we go back after three months, and what if you have cancer? It will already be worse by then.” – Woman, 49 years*

While some women reported that it took months before they received their test results, others even mentioned that they never received them, which of course hampers their trust in the health system. The HPs confirmed that indeed sometimes samples and/or results do get lost and that some women, therefore, have lost interest in cervical cancer screening.

*“I went to the hospital to get screened. I swear I waited one, two, three, four, five, six months for the results, but they never came, and he [the doctor] told me that he is going to take another sample.” – Woman, 35 years*

In general, the women showed little trust in the quality of health services; for example, some women were afraid of getting sick or becoming infected during screening. Some participants had actually experienced bleeding after having a Pap smear; therefore, they suspected that the material used in the procedure was not sterilized well. One participant even mentioned experiencing symptoms of a sexually transmitted infection after the screening. Unclean examination rooms also contributed to this general feeling of mistrust.

*“I have been very suspicious of having to lie down and spread my legs on a stretcher where the sheet shows that it is completely dirty. There were hairs and black dots from the previous patient.” – Woman, 50 years*

With regard to the quality of the test, the HPs also added that false negative results have made them lose trust in the sensitivity of the Pap smear.

*“She was getting a Pap smear every year, and has had over 15 or 20 or 30 tests, or more, and … the next year she died with advanced cancer, and there is nothing that can be done.” – Medical doctor, 50 years*

The HPs felt that this misinterpretation of Pap smears is caused by a lack of training of HPs in either obtaining or managing the samples, or due to poor reading skills of pathologists, resulting in a high number of false negatives.

*“Doctors, not all of them, don’t know how to take a Pap smear. As the doctor said [referring to another participant], it is true, and we have seen here [in the hospital] that the Pap smear is poorly taken, poorly fixed and also poorly analysed.” – Medical doctor, 37 years*

Finally, the HPs considered that there is in general a lack of investment in high-quality cervical cancer screening services. They mentioned that when additional examinations, such as HPV detection or colposcopy, are required, they need to refer patients to specialized health centres, as most health centres do not offer these services. Many of those patients then get lost to follow-up, since there is no system in place to manage or keep track of them at community health centres.

*“I think we fall short in the treatment. We do a Pap smear and find an abnormality, so we need to do an HPV test or we need colposcopy and biopsy, so we send them [the patients] to another place and lose track of the patients.” – Medical doctor, 40 years*

#### Facilitators and suggestions for improvement

The women and HPs identified some options that have shown good results in improving access to health services. For example, both groups mentioned that mobile units can improve access to cervical cancer screening by removing barriers such as distance to health centres or lack of time.

*“Mobile units that offer Pap smears, because, for example, women want to get screened, but because they live far away, first comes the economic aspect, second the time.” – Woman, 33 years*

Reducing the cost of getting screened was also considered important to increase uptake, especially in poor communities where women do not have access to the public health system.

*“Any test that is recommended in the communities should be free of charge, because if people already hear that there is no cost, they will go [for screening].” – Nurse, 42 years*

Nurses also suggested that health centres could dedicate 1 day per week to Pap smears, to increase uptake and reduce waiting times.

*“Health centres should do Pap smears at least once a week; on that day Pap smears are taken for all patients who want one.” – Nurse, 59 years*

They also proposed linking cervical cancer screening with other health services, to increase uptake. They also suggested offering it opportunistically when women come in for other reasons, such as for pre-surgical examinations.

*“On the surgery floor, before they go to have breast surgery or whatever, first thing you do is take a Pap smear.” – Nurse, 50 years*

Furthermore, the HPs recommended using an appointment card to remind women when the next Pap smear should be done.

*“When the Papanicolaou result is given, the next Pap test could be scheduled and written down on a card so that the patient does not miss her appointment and returns.” – Nurse, 35 years*

Some nurses already applied a more active follow-up strategy by calling women who are lost to follow-up.

*“We started calling patients who get lost. People leave us their numbers.” – Nurse, 54 years*

In addition to these rather practical facilitators, the women also emphasized that HPs’ kindness and confidence in HPs would encourage them even more to go for cervical cancer screening. They pointed out that improved communication skills of physicians would contribute to increasing uptake of Pap smears.

*“The respect and explanation that the doctor gives before the procedure. No matter if it is a man or a woman, it’s just that the doctor should always try ... to explain well.” – Woman, 35 years*

### Policy level

#### Barriers

Barriers to cervical cancer screening at policy level were mainly mentioned by the HPs. In their opinion, the national strategy for cervical cancer prevention is not well known by HPs in the field and therefore poorly implemented at operational level. Physicians added that there is no effective health promotion policy, including for cancer screening, which leads to poor knowledge among the population.

*“There is some policy on cervical cancer, but in practice it has not been used as it should be.” – Medical doctor, 78 years*

*“Many of the patients have not been adequately sensitized regarding early detection, and there are no government screening programmes either.” – Medical doctor, 28 years*

Moreover, the HPs mentioned that the current evaluation system inhibits cervical cancer prevention: they are expected to take a certain number of Pap smears per month, rather than being incentivized to reach under-screened women. As a result, some women are over-screened to reach numeric goals, while a large group of women are never invited.

*“We do cytology or Pap smears among the same women ... We can't see the ones who live further away [never-screened women], because all we want is to meet the three or six patients they ask of us [on the target list] at the end of the month.” – Medical doctor, 37 years*

#### Facilitators and suggestions for improvement

Both the women and HPs believed they cannot influence political decisions, but some suggestions were made to improve cervical cancer screening programmes. First, all participants mentioned that national media campaigns could help to encourage women to get screened, by providing information on preventive medicine and how and where they can get screened for cervical cancer.

*“Campaigns, so that the population knows about the exam [cervical cancer screening] or what they have to do in general.” – Medical doctor, 26 years*

Women also suggested that offering screening in the workplace could remove various barriers such as limited time and a lack of engagement.

*“I can tell you that I have done it [getting screened] periodically, because I worked in a public company, and they made us [get screened] practically every year.” – Woman, 58 years*

Others even mentioned that the government should set up health programmes in which certain other benefits are conditional on getting screened. Even mandatory screening was considered an option, with a Pap smear a prerequisite for obtaining certain permissions at government level. In addition, some women had the experience of being screened in the workplace through occupational health programmes. Those initiatives reduce absence from work and the need to ask for permission to seek medical attention.

*“It should be a programme that motivates [women to take up screening] and gives conditions and permission [to get screened at work]. It should be an obligation to take these exams annually.” – Woman, 50 years*

## Discussion

The aim of this research was to identify barriers and facilitators of cervical cancer screening in Cuenca, Ecuador, from the point of view of HPs and under-screened women. The socio-ecological model applied allows different levels of interactions to be identified as causes of adherence to cervical cancer screening. HPs mainly reported barriers and facilitators at policy and individual levels, while the women perceived the limited access to and poor quality of health services as the main problems (organizational level).

Even if there were a robust cervical cancer screening programme in place in Ecuador that offers free screening, women still face barriers that impede full adherence to routine screening. These barriers are a lack of time to attend a medical consultation, and practical difficulties in accessing health centres (physical distance). These limitations were also reported [31]. Strategies that allow women to access screening at their workplace [31–33], the use of mobile medical units in remote areas and the use of portable point of care devices for HPV detection could be effective measures to overcome those obstacles [34–38, 28, 39].

Another barrier at individual level for women is the embarrassment and the fear of pain during a pelvic examination. Given that a lack of privacy prevents them from attending regular screening [40, 41], self-administered tests might be preferred over samples taken by a health care provider and might reduce the fear of pain [42–45].

Additionally, clear communication and empathy among HPs could decrease women’s fear and even overcome the current resistance to being treated by a male HP. This requires adequate training in physician–patient communication and could improve the trust in health services in general, thereby increasing the uptake of cervical cancer screening [46–49].

Similar to other publications, we found that mass media campaigns on cervical cancer prevention would be an important facilitator to improve the uptake of screening [31, 50]. These campaigns increase women’s knowledge and foster a culture of preventive health care in the community [17, 46, 51]. Improved knowledge would also help tackle myths and stigma about this illness and screening [42, 50], strengthen family support to attend screening [52], and empower women to overcome *machismo* that could limit their access to screening [17, 53].

However, the cost of maintaining a mass social media campaign can be high. As such, small-scale health promotion activities at local level should also be considered. The women who participated in the study proposed involving community leaders [17, 20] and incorporating lectures on cancer prevention in adolescents’ sexual and reproductive health education. In health centres, screening could be promoted in the waiting rooms, it could be offered during medical consultations not related to cancer prevention, or health centres could organize screening on a fixed day each week.

For Ecuador, changing from opportunistic screening to an organized programme with direct invitations sent to all eligible women would most likely increase uptake of screening, as shown in other settings [28, 36–39]. Moreover, for the programme to be effective, it should be based on up-to-date national guidelines and protocols, which are currently lacking (national guidelines have not been updated since 2015 in Ecuador). The World Health Organization now proposes that instead of screening women every 3 years using a test with low sensitivity (Pap smear), high-sensitivity tests such as HPV DNA detection tests should be used for primary screening to reduce the burden of repeated screening (i.e. by screening women at least twice during their lifetime, rather than every 3 years). The use of HPV DNA tests as primary screening for cervical cancer has shown advantages in terms of cost-effectiveness due to their higher sensitivity compared with Pap smear and the possibility of self-testing [54–57]. Ecuador should consider the use of HPV DNA tests, as this could reduce the time and effort that both women and health staff have to invest in cervical cancer screening. However, other barriers still have to be addressed to reap the benefits of HPV DNA testing and self-testing [58, 59].

Indeed, improving access to health services will remain crucial. Our study found that it takes a long time for women to obtain an appointment, get screened, receive the results and be referred to a more specialized care centre, if necessary. Even if more sensitive screening tests were used, the structural barriers that cause these delays would need to be addressed. Therefore, the quality of health services should be monitored. A pilot study in Colombia considered that the maximum time between obtaining an appointment and receiving the test results should not exceed 120 days, and the maximum time to provide specific treatment (if required) should not exceed 60 days [31, 33, 60, 61]. Standardizing and reducing the length of each step in the cervical cancer screening cascade could improve the effectiveness of screening and treatment, as well as the satisfaction of women and HPs. Furthermore, instead of evaluating screening programmes based on the number of women screened, the proportion of under-screened women should be monitored as a way to measure performance.

HPs consider that the implementation of a comprehensive cervical screening programme requires resources that are usually not available in low- and middle-income countries. However, the promotion of strategic alliances at the local level, joining public and private resources and efforts, can fortify cervical cancer prevention [17, 53].

### Limitations

The main limitation of this study is that the results cannot be extrapolated to different contexts: they reflect the current reality of women and HPs in the southern part of Ecuador only. Another limitation is that the study mainly reports the point of view of under-screened rather than never-screened women, as all participants had had at least one Pap smear during their lifetime. The reasons for declining cervical cancer screening among never-screened women should be explored in future research.

## Conclusions

The aim of this research was to understand barriers and facilitators of cervical cancer screening among under-screened women and HPs in Ecuador. The HPs who participated in the study reported the main barriers to be the lack of a structured screening programme, low investment in health – and especially in preventive care – cultural patterns at community level linked with myths and *machismo*, and a lack of knowledge. On the other hand, the women interviewed considered the main barriers to be the long waiting times, inadequate patient–physician communication and the perception of Pap smears as a painful and embarrassing procedure.

In terms of facilitators, the HPs proposed that implementing campaigns with community participation would increase awareness about cervical cancer, health literacy and adherence to cervical cancer screening. From the women’s point of view, ensuring fast and friendly access to quality health care and information about prevention will increase women’s health empowerment and improve uptake of medical examinations.

To increase cervical cancer screening, the perspectives of the HPs and the women interviewed should be taken into account, and the quality of services should be continuously monitored. In addition, innovative techniques such as self-administered tests could increase the uptake of screening.

## Data Availability

The datasets generated and analysed during the current study are not publicly available because the focus groups contain sensitive, personal data and the transcripts include the names of participants. The informed consent grants the confidentiality of the participants’ data. However, the datasets are available from the corresponding author on request on reasonable grounds.

## Abbreviations

FGD: Focus group discussion
HP: Health professional
HPV: Human papillomavirus
SOLCA: Sociedad de Lucha Contra el Cáncer
UC-COBIAS: Universidad de Cuenca Comité de Bioética de las áreas de la Salud (University of Cuenca Committee of Bioethics in Health Science Areas)

## References

[1] Sung H, Ferlay J, Siegel RL, Laversanne M, Soerjomataram I, Jemal A (2021). Global cancer statistics 2020: GLOBOCAN estimates of incidence and mortality worldwide for 36 cancers in 185 countries. CA Cancer J Clin.

[2] World Health Organization (2021). WHO guideline for screening and treatment of cervical pre-cancer lesions for cervical cancer prevention.

[3] Canfell K, Kim JJ, Brisson M, Keane A, Simms KT, Caruana M (2020). Mortality impact of achieving WHO cervical cancer elimination targets: a comparative modelling analysis in 78 low-income and lower-middle-income countries. Lancet.

[4] McGraw SL, Ferrante JM (2014). Update on prevention and screening of cervical cancer. World J Clin Oncol.

[5] International Agency for Research on Cancer. Ecuador Fact sheets 2020: IARC. p. 2021. Available from: https://gco.iarc.fr/today/data/factsheets/populations/218-ecuador-fact-sheets.pdf Accessed 2 Oct 2022

[6] Bruni L, Albero G, Serrano B, Mena M, Gómez D, Muñoz J, Bosch FX, de Sanjosé S (2019). Human Papillomavirus and Related Diseases Report Ecuador.

[7] Vega, B. Prevalencia de cáncer de cuello uterino en el Ecuador y estrategias para su reducción. Facultad de Ciencias Médicas Universidad de Cuenca. 2012;30(1):45–51. Available from: http://dspace.ucuenca.edu.ec/handle/123456789/20387. Accessed 2 Oct 2022.

[8] Ministerio de Salud Pública del Ecuador. Estrategia Nacional para la prevención del Cáncer en el Ecuador 2017. MSP; 2017. Available from: https://aplicaciones.msp.gob.ec/salud/archivosdigitales/documentosDirecciones/dnn/archivos/ac_0059_2017.pdf Accessed 11 Nov 2021.

[9] Ministerio de Salud Pública del Ecuador. Plan Nacional de Salud Sexual y Salud Repoductiva 2017–2021. MSP; 2017. Available from: https://ecuador.unfpa.org/sites/default/files/pub-pdf/PLAN%20NACIONAL%20DE%20SS%20Y%20SR%202017-2021.pdf Accessed 11 Nov 2021.

[10] Ministerio de Salud Pública del Ecuador. Esquema de vacunación Ecuador 2019. MSP; 2019. Available from: https://www.salud.gob.ec/wp-content/uploads/2020/01/ESQUEMA-DE-VACUNACIO%CC%81N.DIC_.2019.ok_.pdf Accessed 11 Nov 2021.

[11] Ministerio de Salud Pública del Ecuador. Protocolos con evidencia para la detección oportuna del cáncer de cuello uterino. MSP; 2015. Available from: https://webcache.googleusercontent.com/search?q=cache: FxdiESuIbPYJ:https://aplicaciones.msp.gob.ec/salud/archivosdigitales/sigobito/tareas_seguimiento/1614/protocolos_cancer_c%25C3%2589rvico_uterino._13_revision__borrador.-1.doc+&cd=1&hl=es&ct=clnk&gl=ec Accessed 11 Nov 2021.

[12] Instituto Nacional de Estadísticas y Censos. Anuario de camas y egresos hospitalarios. INEC; 2019. Available from: https://www.ecuadorencifras.gob.ec/camas-y-egresos-hospitalarios/ (accessed 11 November 2021).

[13] World Health Organization (2020). Global strategy to accelerate the elimination of cervical cancer as a public health problem.

[14] Benard VB, Royalty J, Saraiya M, Rockwell T, Helsel W (2015). The effectiveness of targeting never or rarely screened women in a national cervical cancer screening program for underserved women. Cancer Causes Control.

[15] Instituto Nacional de Estadísticas y Censos. Encuesta Nacional de Salud y Nutrición 2018. INEC, Quito, Ecuador; 2018. Available from: https://www.ecuadorencifras.gob.ec/documentos/web-inec/Estadisticas_Sociales/ENSANUT/ENSANUT_2018/Principales%20resultados%20ENSANUT_2018.pdf Accessed 11 Nov 2021.

[16] Siegel RL, Miller KD, Jemal A (2019). Cancer statistics, 2019: Cancer statistics, 2019. CA Cancer J Clin.

[17] Nugus P, Désalliers J, Morales J, Graves L, Evans A, Macaulay AC (2018). Localizing global medicine: challenges and opportunities in cervical screening in an indigenous community in Ecuador. Qual Health Res.

[18] Soneji S, Fukui N. Socioeconomic determinants of cervical cancer screening in Latin America. Rev Panam Salud Publica 2013;33(3):174–182. 10.1590/s1020-49892013000300003. Available from: Accessed 2 Oct 2022. 10.1590/s1020-49892013000300003 PMC3724344 23698136

[19] Corral F, Cueva P, Yépez J, Montes E (1996). Limited education as a risk factor in cervical cancer. Bull Pan Am Health Organ.

[20] Godoy Y, Godoy C, Reyes J (2016). Social Representations of Gynecologic Cancer Screening Assessment a Qualitative research on Ecuadorian women. Rev Esc Enferm USP.

[21] O’Cathain A, Hoddinott P, Lewin S, Thomas KJ, Young B, Adamson J (2015). Maximising the impact of qualitative research in feasibility studies for randomised controlled trials: guidance for researchers. Pilot Feasibility Stud.

[22] Nyumba T, Wilson K, Derrick CJ, Mukherjee N (2018). The use of focus group discussion methodology: insights from two decades of application in conservation. Geneletti D, editor. Methods Ecol Evol.

[23] Tong A, Sainsbury P, Craig J (2007). Consolidated criteria for reporting qualitative research (COREQ): a 32-item checklist for interviews and focus groups. Int J Qual Health Care.

[24] Lindgren B-M, Lundman B, Graneheim UH (2020). Abstraction and interpretation during the qualitative content analysis process. Int J Nurs Stud.

[25] Linares ET, Vilariño CS, Villas MA, Álvarez-Dardet SM, López MJL (2002). El Modelo Ecológico de Bronfrenbrenner Como Marco Teórico de la Psicooncología. An psicol.

[26] Rodriguez SD, Vanderford NL, Huang B, Vanderpool RC (2018). A social-ecological review of cancer disparities in Kentucky. South Med J.

[27] Daley E, Alio A, Anstey EH, Chandler R, Dyer K, Helmy H (2011). Examining barriers to cervical cancer screening and treatment in Florida through a socio-ecological lens. J Community Health.

[28] Urrutia M-T, Araya A, Jaque M-F (2017). Why do Chilean women choose to have or not have pap tests?. J Obstet Gynecol Neonatal Nurs.

[29] Colorafi KJ, Evans B (2016). Qualitative descriptive methods in health science research. HERD.

[30] Azungah T (2018). Qualitative research: deductive and inductive approaches to data analysis. QRJ.

[31] Binka C, Nyarko SH, Awusabo-Asare K, Doku DT (2019). Barriers to the uptake of cervical cancer screening and treatment among rural women in Ghana. Biomed Res Int.

[32] Coughlin SS, Caplan LS, Lawson HW (2002). Cervical cancer screening in the workplace. Research review and evaluation. AAOHN J.

[33] Agurto I, Bishop A, Sánchez G, Betancourt Z, Robles S (2004). Perceived barriers and benefits to cervical cancer screening in Latin America. Prev Med.

[34] Swaddiwudhipong W, Chaovakiratipong C, Nguntra P, Mahasakpan P, Tatip Y, Boonmak C (1999). A mobile unit: an effective service for cervical cancer screening among rural Thai women. Int J Epidemiol.

[35] Okunade KS, Soibi-Harry A, John-Olabode S, Adejimi AA, Allsop MJ, Onyeka TC (2021). Impact of mobile technologies on cervical cancer screening practices in Lagos, Nigeria (mHealth-cervix): a randomized controlled trial. JCO Glob Oncol.

[36] Austad K, Chary A, Xocop SM, Messmer S, King N, Carlson L (2018). Barriers to cervical cancer screening and the cervical cancer care continuum in rural Guatemala: a mixed-method analysis. J Glob Oncol.

[37] Zorogastua K, Erwin D, Thelemaque L, Pulley L, Jandorf L (2016). Intrinsic factors of non-adherence to breast and cervical cancer screenings among Latinas. J Racial Ethn Health Disparities.

[38] Paulauskiene J, Ivanauskiene R, Skrodeniene E, Petkeviciene J (2019). Organised versus opportunistic cervical cancer screening in urban and rural regions of Lithuania. Medicina (Kaunas).

[39] Nagendiram A, Bougher H, Banks J, Hall L, Heal C (2020). Australian women’s self-perceived barriers to participation in cervical cancer screening: a systematic review. Health Promot J Austr.

[40] Baezconde-Garbanati L, Agurto I, Gravitt PE, Luciani S, Murphy S, Ochoa C (2019). Barriers and innovative interventions for early detection of cervical cancer. Salud Publica Mex.

[41] Black E, Hyslop F, Richmond R (2019). Barriers and facilitators to uptake of cervical cancer screening among women in Uganda: a systematic review. BMC Womens Health.

[42] Jentschke M, Lehmann R, Drews N, Hansel A, Schmitz M, Hillemanns P (2020). Psychological distress in cervical cancer screening: results from a German online survey. Arch Gynecol Obstet.

[43] Cadman L, Waller J, Ashdown-Barr L, Szarewski A (2012). Barriers to cervical screening in women who have experienced sexual abuse: an exploratory study. J Fam Plann Reprod Health Care.

[44] Sy AU, Hernandez BY, Tareg A, Reichhardt M, Buenconsejo-Lum L (2017). Acceptability and feasibility of a community based participatory research project comparing cytology and urine HPV DNA testing for cervical cancer screening in Yap, Federated States of Micronesia. Cancer Epidemiol.

[45] Rosenbaum AJ, Gage JC, Alfaro KM, Ditzian LR, Maza M, Scarinci IC (2014). Acceptability of self-collected versus provider-collected sampling for HPV DNA testing among women in rural El Salvador. Int J Gynaecol Obstet.

[46] Richman AR, Troutman JL, Torres E (2016). Experiences of cervical cancer survivors in rural eastern North Carolina: a qualitative assessment. J Cancer Educ.

[47] Olaza-Maguiña AF, De la Cruz-Ramirez YM (2019). Barriers to the non-acceptance of cervical cancer screenings (pap smear test) in women of childbearing age in a rural area of Peru. Ecancermedicalscience.

[48] Liebermann EJ, VanDevanter N, Shirazian T, Frías Gúzman N, Niles M, Healton C (2020). Barriers to cervical cancer screening and treatment in the Dominican Republic: perspectives of focus group participants in the Santo Domingo area. J Transcult Nurs.

[49] Getachew S, Getachew E, Gizaw M, Ayele W, Addissie A, Kantelhardt EJ (2019). Cervical cancer screening knowledge and barriers among women in Addis Ababa, Ethiopia. PLoS One.

[50] Akinlotan M, Bolin JN, Helduser J, Ojinnaka C, Lichorad A, McClellan D (2017). Cervical cancer screening barriers and risk factor knowledge among uninsured women. J Community Health.

[51] Jolidon V, De Prez V, Willems B, Bracke P, Cullati S, Burton-Jeangros C (2020). Never and under cervical cancer screening in Switzerland and Belgium: trends and inequalities. BMC Public Health.

[52] Vrinten C, Gallagher A, Waller J, Marlow LAV (2019). Cancer stigma and cancer screening attendance: a population based survey in England. BMC Cancer.

[53] Galvão JR, de Almeida PF, Santos AMD, Bousquat A (2019). Percursos e obstáculos na Rede de Atenção à Saúde: trajetórias assistenciais de mulheres em região de saúde do Nordeste brasileiro. Cad Saude Publica.

[54] Ogilvie GS, van Niekerk D, Krajden M, Smith LW, Cook D, Gondara L (2018). Effect of screening with primary cervical HPV testing vs cytology testing on high-grade cervical intraepithelial neoplasia at 48 months: the HPV FOCAL randomized clinical trial. JAMA.

[55] Sankaranarayanan R, Budukh AM, Rajkumar R (2001). Effective screening programmes for cervical cancer in low- and middle-income developing countries. Bull World Health Organ.

[56] Philp L, Jembere N, Wang L, Gao J, Maguire B, Kupets R (2018). Pap tests in the diagnosis of cervical cancer: help or hinder?. Gynecol Oncol.

[57] Vega Crespo B, Neira VA, Ortíz Segarra J, Rengel RM, López D, Orellana MP (2022). Role of self-sampling for cervical cancer screening: diagnostic test properties of three tests for the diagnosis of HPV in rural communities of Cuenca, Ecuador. Int J Environ Res Public Health.

[58] Shin HY, Lee B, Hwang SH, Lee DO, Sung NY, Park JY (2019). Evaluation of satisfaction with three different cervical cancer screening modalities: clinician-collected Pap test vs. HPV test by self-sampling vs. HPV test by urine sampling. J Gynecol Oncol.

[59] Bautista-Valarezo E, Vega Crespo B, Maldonado-Rengel R, Espinosa ME, Neira VA, Verhoeven V (2022). Knowledge and perceptions about cervical cancer and HPV screening in women in rural areas of Ecuador: a qualitative research study. Int J Environ Res Public Health.

[60] Buss LF, Levi JE, Longatto-Filho A, Cohen DD, Cury L, Martins TR (2021). Attendance for diagnostic colposcopy among high-risk human papillomavirus positive women in a Brazilian feasibility study. Int J Gynaecol Obstet.

[61] Sardi A, Orozco-Urdaneta M, Velez-Mejia C, Perez-Bustos AH, Munoz-Zuluaga C, El-Sharkawy F (2019). Overcoming barriers in the implementation of programs for breast and cervical cancers in Cali, Colombia: a pilot model. J Glob Oncol.

